# Supplementary material for: Chromothripsis during telomere crisis is independent of NHEJ, and consistent with a replicative origin
Source: Genome Res. 2019 May;29(5):737–49. doi: 10.1101/gr.240705.118 (PMC6499312; doi:10.1101/gr.240705.118)
Supplement: Supplemental Material [file supp_gr.240705.118_Supplemental_file_1.zip › contigs/annotated_contigs/DB51/contig.2.DB51_length_787_mean_cov_8.51588310038.docx]

**DB51_length_787_mean_cov_8.51588310038**

```
CAGGTACATGCACTCAACAGCATGTCCATGAACCCTGTTCTCTTCTGGAGGGTATACGTGGCAGAAGCTCTGGCTCTTTCACATCAAGA
 >chr7:143154753-143155216 + E=1e-263
AGGATCTTAAATTCTGGAGCAATGGCTCATACCTATAATCCCAGCACTTTGAGAGGCTGATGCAGGCAGATCGCTTGAGTCCAGGAGTT

CCAGGCTAGTCTGGGCAACATAGCAAAATCCCATCTCTACAAAAAGTACAATTAGCCAGGTGTGGTCGCATGCGCCTGTGGTCCCAGCT

ACTCAGGAGGCTGAGAAGGGAGGATTGCTTGAGCCCGGGGAGGTCAAGGCTGCAGTGAGCTGTGATCACACCACTGCACTCCAGTCTGG

GTGACAGAGTGCAGTCCCTGTCCTAAAATCAACAGCATTCTCATTCGAGTAAAAAATAATTAAACCTTGAGATATTTTCCTCTGCTTTC

CTATGCTCTCCATTCA|CA|AAAAGGCAAAAGGCAAAAGGGCAAAAGGCATGTGGGCACTCCCTTCAATTTCTTTTGTAAGTGCACTGA
                 >chr7:143156586-143156912 + E=3e-184
TCTGTTCATGAGGCTCCACCCTCAAGAATGGATTAGTGCTCTTATAAAATAGACTTAATCACTTCCCCAAAGGCCCAGTCTCTTAATGT

GGTTGCATTGGCAATTAGGTTTCAACATGTGAATTTTGAGGGACACATTTAAACCACAGCAATATCACAAAGAATTCATTAGTTTTAAA

ACTTAAATATTATTTTTGATAATACATGATTATTGCAGAAAATATGGAAAACAGACTATTATTAAAAAGTTAAAAAT
```
